# Research on gradient aging behaviors of asphalt and decay characteristics of its properties under complex UV radiation conditions based on component diffusion and aging kinetics theory

**DOI:** 10.1371/journal.pone.0329496

**Published:** 2025-08-05

**Authors:** Hengbin Liu, Zhongnan Tian, Ying Fang, Zhengqi Zhang, Yulong Zhao, Peng Hu, Kun Wang

**Affiliations:** 1 Shandong Jiaotong University, Jinan, Shandong, China; 2 Shandong Key Laboratory of Technologies and System for Intelligent Construction Equipment, Jinan, Shandong, China; 3 Hebei Provincial Communications Planning, Design and Research Institute Co., Ltd, Shijiazhuang, Hebei, China; 4 School of Civil Engineering and Architecture, Hubei University of Arts and Science, Xiangyang, Hubei, China; 5 Hubei Key Laboratory of Vehicle-infrastructure Cooperation and Traffic Control, Xiangyang, Hubei, China; 6 Chang’an University, Xi’an, Shaanxi, China; University of Salerno: Universita degli Studi di Salerno, ITALY

## Abstract

To gain a more comprehensive understanding of UV aging process of base asphalt and SBS polymer modified asphalt, the gradient aging behaviors of asphalt and decay characteristics of their properties under different UV radiation conditions were investigated. Initially, different types of asphalt film specimens were subjected to UV aging test under complex radiations. Then, the aged asphalt samples were stripped layer by layer through the stratified elution method. And frequency scanning test for asphalt from different layers were carried out. Subsequently, the gradient UV aging behaviors and aging depth of asphalt were analyzed with G*, G*AI and diffusion coefficient *D*. Finally, UV aging kinetics equations of asphalt under different UV radiations were established to analyze the decay characteristics of its properties. Results show that UV aging process of asphalt exists the significant gradient aging distribution phenomenon, which starts from the surface of asphalt and gradually develops to the deeper layer of asphalt. Relationship between the aging depth of asphalt and aging times be characterized by a two-stage model, with the logarithm model in the early stage and the linear stage in the later stage. Enhancement of radiation intensity and aging temperature will both lead to the increase in the *D* values of asphalt, thus making the UV aging gradient behaviors of asphalt more obvious. Results of aging kinetics equations show that UV aging process of asphalt also follows the two-stage reaction phenomenon. And aging time of 24 hours can be set as the criteria to distinguish between the two-stage reaction zones. Meanwhile, enhancement of radiation intensity and aging temperature can also both lead to the increase in the aging reaction rate of asphalt. By contrast, radiation intensity has a greater effect on the UV aging reaction rate of asphalt than aging temperature, indicating that radiation intensity dominates the main effect in the degradation process of asphalt’s UV aging properties.

## 1. Introduction

Asphalt pavement acts as an important role in the construction of transportation infrastructure, and has become the main type of pavement structure [1,2]. However, as the structure layer in direct contact with the external environment, asphalt pavement is prone to aging under the complex service environmental conditions, such as high or low temperature, existence of water, ultraviolet radiation and emissions from binders [3–5]. One of the main reasons causing the damage of road performance for asphalt pavement is the aging of asphalt. As the binders of asphalt mixture, asphalt plays the role in bonding the loose aggregates and fillers [6,7]. Oxidation and other chemical changes that occur in asphalt upon aging cause hardening and embrittlement [8], and further reduce the adhesion of interface between asphalt and aggregates [9]. Thus, under the coupling effect of heavy loading, some diseases, such as rutting, cracking and peeling, appear on asphalt pavement, which seriously affects its road performance and service life [10]. Generally, aging of asphalt can be divided into three types according to the contributing factors, including thermal oxidation aging, ultraviolet (UV) aging and water aging [11]. Currently, scholars have focused more attention on the thermal oxidation aging of asphalt while neglecting the UV aging and water aging [12]. However, it is undeniable that the above two types of aging will also affect the road properties of asphalt, especially the conditions of UV radiation. And this has been confirmed in many studies and practical engineering [13–15].

Due to the different action conditions, there are significant differences in the processes and mechanism of thermal and UV aging for asphalt. As for its thermal oxidation aging, the main conditions affecting aging process are temperature and oxygen. On one hand, the light components (such as saturates and aromatics) in asphalt will evaporate at higher temperature, causing an imbalance in the proportion of asphalt components. On the other hand, high temperature thermal decomposition can cause the chemical bonds in asphalt to break, and then under the presence of oxygen, the broken chemical bonds will undergo the oxidative addition reactions, thus polar oxygen-containing functional groups, such as Carbonyl and sulfoxide groups. While the UV aging is triggered by the absorption of energetic UV light by groups in the molecule and their conversion from the ground state to the excited state, which leads to the breaking of chemical bonds [16]. Meanwhile, effects of UV aging are concentrated mostly at the surface of asphalt [17]. However, due to the influence of diffusion and fusion, the UV aging depth of asphalt gradually deepens with the extension of aging time, which presents the obvious gradient distribution phenomenon, thus causing a bigger difference from thermal oxidative aging of asphalt.

Besides, different from the thermal aging, UV aging behavior of asphalt will be affected by various factors, such as UV radiation intensity, aging temperature, radiation time, and asphalt types [18]. To explore the decay characteristics of asphalt’s properties during UV aging process, researchers have conducted the UV aging simulation test under different radiation conditions. For example, Ju [19] et al. investigated the influence of the variable intensity ultraviolet radiation on road properties of asphalt, and concluded that the variable intensity UV aging conditions induced a much larger aging effect for UV-aged asphalt than constant intensity UV aging conditions. Wu [13] et al. has also found that the increasing of UV radiation intensity can significantly promote the UV aging degree of asphalt. And Zeng [20] et al. explored the effect of aging temperature on the UV aging degree of asphalt. In their study, they conducted three temperature test conditions, including 30°C, 50°C and 70°C, and found that when aging temperature exceeded 70°C, it has a significant promoting effect on the UV aging degree of asphalt. Liu [11] et al. studied the effect of UV radiation times on asphalt’s properties based on the index of G*AI, and found that with the extension of aging time, UV aging rate of asphalt shows a trend of rapid growth firstly and then to be stable. Moreover, they also employed response surface methodology (RSM) to investigate the significant impact of different UV radiation conditions on asphalt properties, and found that during the UV aging process of asphalt, effect of asphalt film on the UV aging rate of asphalt is the largest, while that of aging temperature is the smallest. Pei [21] et al. analyzed the micro characteristics for different kinds of asphalt under UV aging conditions, and found that compared with the asphalt grade, oil source of asphalt exerted a greater impact on micro characteristics of asphalt. From the relevant researches, it can be clearly seen that UV aging characteristics of asphalt will be influenced by multiple factors, which further leads to properties of asphalt after UV aging variety more difficultly.

In addition, due to the limited penetration of ultraviolet light into the asphalt film, UV aging process of asphalt exists the significant gradient aging distribution phenomenon, which results in the complexity of its aging process. For example, research of Zeng [22] et al. indicated that the only about 0 ~ 5μm deep asphalt samples would be directly radiated by ultraviolet light, while the deeper aged asphalt is mainly caused by the diffusion process of the surface aged asphalt, and when the UV aging time reached to 10 days, the diffusion depth of UV aged asphalt could be nearly about 2200μm. Chen [23] et al. found that under the UV aging conditions, as the vertical position of asphalt layer deepens, content of large molecules in the asphalt gradually decreases, while that of the small molecules gradually increases, confirming that the gradient aging distribution phenomenon occurs in the UV aging process of asphalt. Hu [24] et al. studied the relationship between the UV aging time and penetration depth of UV radiation into asphalt, and found that when UV aging time reached to 10 hours, the penetration depth of UV radiation was less than 13μm. Considering the complexity of UV radiation conditions, Li [25] et al. explored the gradient aging behaviors of asphalt aged by ultraviolet light with various intensities, and found that under the UV radiation conditions with higher intensity, gradient aging behaviors of asphalt is more significant. Overall, exploring the gradient UV aging distribution behavior of asphalt is of great significance for understanding the decay characteristics of asphalt properties and its aging mechanism, thus helping to propose the effective measures to improving the aging resistance. However, the previous studies have mostly focused on the gradient UV aging distribution behavior of asphalt under the single conditions, without considering the influence of other factors. And as mentioned in the previous section, UV aging behaviors of asphalt is significantly affected by the various radiation conditions. Under different UV radiation conditions, decay characteristics of asphalt properties will present different change trend. Therefore, it is not comprehensive enough to explore the gradient UV aging distribution behavior of asphalt under the single conditions. And it must be necessary to consider the effects of complex UV radiation conditions on asphalt’s properties with some advanced methods (such as aging kinetics theory), so as to better understand the UV aging mechanism of asphalt.

Aimed at the limitations of the above researches, purpose of this study is to explore the gradient aging behaviors of asphalt and decay characteristics of its properties under different UV radiation conditions from the macro perspective. To achieve this goal, different types of asphalt (including base asphalt and SBS modified asphalt) were subjected to UV aging test under complex radiation conditions initially. Subsequently, the aged asphalt from different depths were collected layer by layer through stratified elution method, and frequency scanning test was conducted to obtain the G* values of asphalt. On this basis, diffusion coefficient *D* of the surface UV aged asphalt at different layers were calculated through the Fick’s second diffusion law, so as to understand the effect of various radiation conditions on the gradient UV aging behavior of asphalt. Furthermore, taken the G* values of the aged asphalt samples on the surface as index, UV aging kinetics equations of asphalt were established, and the reaction rate constant *k* was obtained to analyze the decay characteristics of asphalt’s properties under UV aging conditions. These findings will provide some reference for understanding the UV aging mechanism of asphalt, thus helping to propose effective anti-aging measures for asphalt.

## 2. Materials and methodology

### 2.1. Materials

Four types of asphalt binders were selected in this study, including two base asphalt samples and two SBS modified asphalt samples, which were named as SK70, SK90, SK70/SBS and SK90/SBS respectively. According to the ASTM testing procedures, the basic technical indexes of asphalt samples were tested, and the results are shown in Table 1.

**Table 1:** Technical indexes of SBS modified asphalt.

Technical indexes	Unit	SK70	SK90	SK70/SBS	SK90/SBS
**Penetration**	0.1 mm	71	82	51	59
**Softening point**	°C	47.5	50.0	65.5	68
**Ductility**	cm	53 (10°C)	40 (10°C)	24 (5°C)	28 (5°C)
**Rotational viscosity (135°C)**	Pa·s	0.475	0.416	1.570	1.510
**Solubility**	%	99.86	99.90	99.20	99.36
**Retained penetration ratio**	%	62.02	68.46	76.33	94.05
**Retained ductility after RTFOT**	cm	11 (10°C)	10 (10°C)	20 (5°C)	23 (5°C)

### 2.2. Methodology

#### 2.1.1. Indoor simulation of UV aging test.

**Fig 1:**
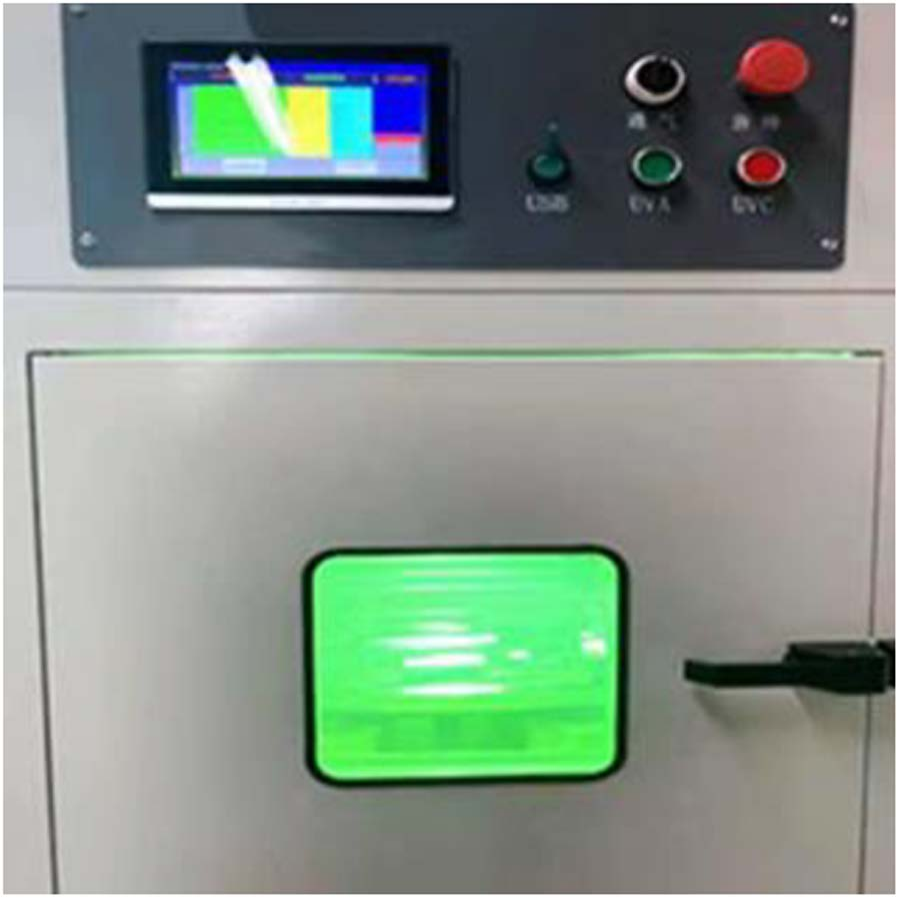
UV aging equipment.

**Fig 2:**
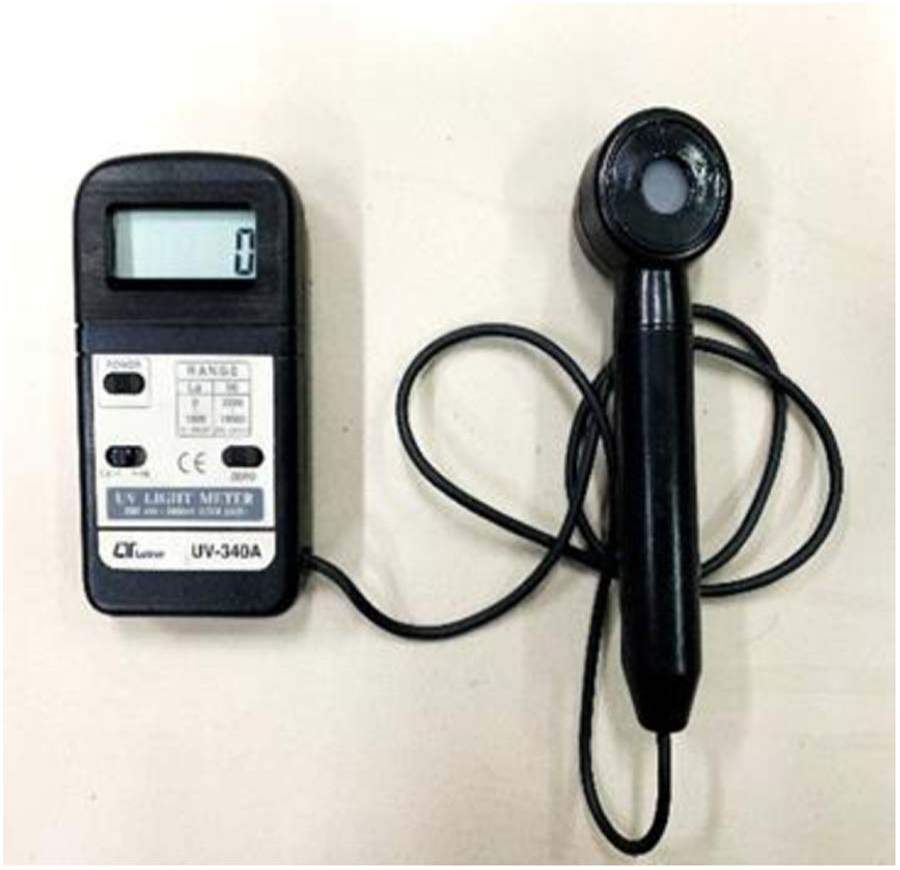
UV light meter.

The UV aging process of asphalt occurs in the service time of pavement, which belongs to the long-term aging process, so Thin Film Oven Test (TFOT) was carried out first to simulate the short-term thermal oxidation aging process [26]. After TFOT test, the aged asphalt samples were put in an UV aging equipment (as shown in Fig.1) to conduct UV aging process. Four groups of UV lamp are installed in the equipment with rotating speed of 10 r/min, and the radiation intensity can be adjusted as needed. To accurately measured the ultraviolet radiation intensity, UV light meter UV-340A (as shown in Fig 2) was utilized. Meanwhile, testing temperature can also be adjusted with the range of 25°C ~ 55°C.

#### 2.1.2. Laboratory UV aging simulation conditions.

Effects of UV aging on asphalt properties include the internal and external factors. Internal factors mainly refer to types of asphalt and its molecular weight. External factors mainly refer to aging temperature, aging time and UV radiation intensity. Therefore, to explore effects of different UV radiation conditions on decay characteristics of asphalt properties, following experimental conditions (as listed in Table 2) were selected in this study.

**Table 2:** Different UV radiation conditions.

Test parameters	Parameter values
**UV radiation intensity**	90 W/m^2^、110 W/m^2^、130 W/m^2^
**UV aging temperature**	30°C、35°C、40°C
**UV aging times**	1h、3h、6h、9h、12h、24h、72h、144h、216h
**Asphalt types**	SK70、SK90、SK70/SBS、SK90/SBS

#### 2.1.3. Layered treatment method for UV aged asphalt samples.

**Fig 3:**
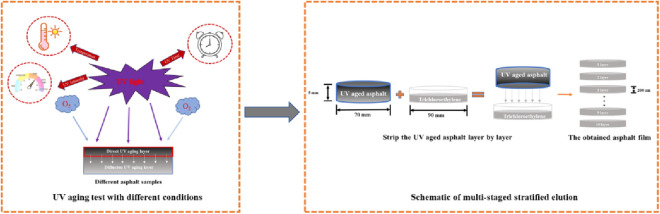
Schematic diagram of UV aging diffusion behavior of asphalt.

Previous studies have shown that UV aging process of asphalt exists the significant gradient aging distribution phenomenon, thus the properties of asphalt at different depths will exhibit different decay characteristics [23,26]. The main reason for the above phenomenon is that UV aging of asphalt exits significant diffusion process. As displayed in Fig.3, to investigate the effects of UV radiation conditions on gradient distribution of asphalt’s UV aging, the stratified elution method was applied to treat the aged asphalt samples under different UV aging conditions in layers [26]. Then DSR test was carried out to obtain G* values at 60°C of the stripped asphalt samples, and diffusion coefficient *D* was calculated to analyze the diffusion behavior of UV aged asphalt. It should be noted that during DSR test, to ensure the scientificity of test data, three repeatability tests were carried out for each samples, and finally the average value is taken as the test result.

**Fig 4:**
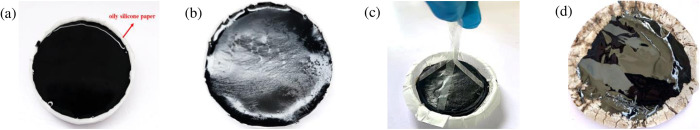
Layered peeling process for UV aged asphalt samples. **(a)** Asphalt sample before UV aging test; **(b)** Asphalt sample after UV aging test; **(c)** Stripping treatment; **(d)** Asphalt film sample.

Fig 4 shows the schematic diagram for stripping the UV aged asphalt samples layer by layer, and the specific process is described as follows:

(1) As shown in Fig.4 (a), before UV aging test for asphalt, the inner wall of sample container was wrapped with a layer of oily silicone paper to prevent it from bonding with the asphalt samples. Then, asphalt was heated to achieve the flow state and poured into the sample container. Considering the requirements of this study, film thickness of asphalt samples should be controlled at about 5000 um. After the samples cooled down, they were placed in the aging equipment to implement UV aging process.
(2) After UV aging test was completed, the aged asphalt sample was taken out and cooled at room temperature at least 2 hours. Then the oily silicone paper wrapped around the out wall of asphalt samples was removed, thus the UV aged samples were obtained, which was displayed in Fig.4 (b).
(3) Before conducting the stripping test on asphalt, a glass dish with the inner diameter of 90 mm was prepared. Then its inner wall was wrapped with a layer of oily silicone paper. And 10 ml trichloroethylene reagent was poured in the dish as the stripping solvent. Subsequently, as shown in Fig.4 (c), the side of aged asphalt sample was fixed with the transparent tape, and then placed it upside down in the trichloroethylene stripping solvent. After the aged asphalt sample was suspended in the solvent for a period of time, the trichloroethylene solution containing the asphalt samples can be obtained.
(4) The oily silicone paper containing trichloroethylene solution was taken out and then placed it in a fume hood. After the trichloroethylene solvent was completely volatilized, UV aged asphalt film was obtained. Then, the oily silicone paper containing aged asphalt samples was put into an oven at 40°C for further drying treatment. It must be noted that for SBS modified asphalt, temperature needs to be raised to 60°C to completely eliminate the effect of trichloroethylene solvent on asphalt properties. After drying treatment, the peeled UV aged asphalt samples was obtained, which was shown in Fig 4 (d). And the mass of asphalt samples was measured for subsequent conversion of asphalt film thickness.

As for studying the diffusion behaviors of asphalt’s UV aging process, the key step is to control the peel thickness of asphalt samples. This step can be processed by controlling the soaking time of asphalt samples in trichloroethylene reagents. Considering requirements of asphalt properties test and peeling thickness of asphalt film, after repeated experiments, the soaking time was controlled at 120 s to ensure that sufficient asphalt samples can be obtained.

And then mass of each layer asphalt samples was measured and the film thickness was calculated according to Equation (1). Based on the aging degree of asphalt at different layers, asphalt sample can be peeled up to 10 times.


$$h=\frac{4m}{\pi d^{2}\rho }$$ (1)

where: *h*--- thickness of film, um; *m*--- quality of film, g; *d*--- diameter of the sample container, cm; *ρ*--- density of asphalt, g/cm^3^.

#### 2.1.4. Calculation process of diffusion coefficient *D.*

Generally, the Fick’s diffusion theory is generally used to describe the relationship between mass transfer flux and concentration gradient in molecular diffusion processes, which includes the Fick’s first diffusion law and Fick’s second diffusion law. Among them, the Fick’s first diffusion law is often used to describe the steady state diffusion process of molecules, and the Fick’s second diffusion law is often used to describe the non-steady state diffusion process of molecules. Asphalt belongs to an organic polymer mixture composed of various substances such as cycloalkanes, alkanes, aromatics, etc. Therefore, as for diffusion process of UV aged asphalt, concentration of the aged asphalt in the diffusion system will vary with the change of diffusion time and diffusion distance. In other words, UV aging diffusion process of asphalt belongs to the non-steady state diffusion process. So Fick’s second diffusion law was selected to explore the UV aging diffusion process of asphalt [27]. Expression of Fick’s second diffusion law is shown in Equation (2).


$$\frac{\partial C}{\partial t}=\frac{\partial }{\partial x}\left(D\frac{\partial C}{\partial x}\right)$$ (2)

Generally, *D* is often approximated as a constant. Thus, Equation (1) can be converted to Equation (3).


$$\frac{\partial C}{\partial t}=D\frac{\partial^{2}C}{\partial x^{2}}$$ (3)

**Fig 5:**
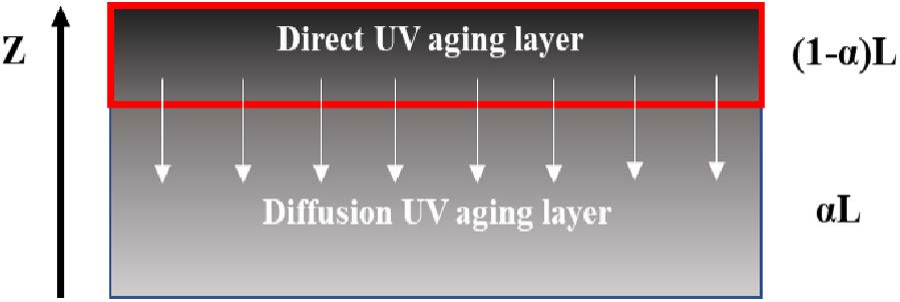
Model of the UV aging diffusion for asphalt.

To solve the *D* conveniently, referring to one-dimensional two-layer diffusion model established by Karlsson [28] et al., which was developed to describe the diffusion pathway of rejuvenators in aged asphalt, a simple model for describing the diffusion process of surfaced UV aged asphalt in the deep un-aged asphalt was constructed. As shown in Fig 5, this model assumes that concentration of the UV aged asphalt layer is c_0_, the total thickness of the aged layer and diffusion layer is L, the thickness of diffusion layer is αL, and that of the aging layer is (1-α)L.

For solving the model conveniently, the following assumptions were made: (1) without considering the issue of asphalt composition, the aging layer and diffusion layer are assumed to be two different asphalt layers. (2) concentration c_0_ of the aged layer’s asphalt is assumed to be a certain value, which does not change with the change of diffusion time. (3) asphalt layer with a thickness of about 200μm is assumed to be an aged asphalt layer under the direct ultraviolet radiation.

Base on the above assumptions, the boundary conditions of the diffusion model in Fig.5 can be described as follows:


c(z,0)=0    0≤z≤L (4)

$$c(z,0)=c_{0}\;\alpha L\leq z\leq L$$ (5)

$$\frac{\partial c(0,t)}{\partial z}=\frac{\partial c(L,t)}{\partial z}=0$$ (6)

After the above conditions were established, the expression of Fick’s second diffusion law (Equation 3) was decomposed into two first-order differential equations, and then the boundary conditions were incorporated into them, thus solving the concentration *c(z, t)* of asphalt in the diffusion layer at different positions and times [29]. And the expression of *c(z, t)* is shown in Equation (7).


$$\begin{array}{c} c(z,t)=(1-\alpha )\cdot c_{0}-\frac{2c_{0}}{\pi }\cdot \sum {\mathrm{\backslash nolimits}}_{n=1}^{\infty }\frac{\sin(\alpha n\pi )}{n}\cdot \cos\left(\frac{n\pi z}{L}\right)e^{{-\left[n(\pi /L)\right]}^{2}Dt} \end{array}$$ (7)

Where: *c(z, t)*---concentration of asphalt at diffusion distance z and diffusion time t; *c*_*0*_---initial concentration of the UV aged layer’s asphalt; *L*--- the total thickness; *n*--- the total number of layers; *α*---scale factor; *D*---diffusion coefficient.

To solve *D*, the theory of composite material was also introduced in this study, whose expression was expressed as Equation (8). According to above equation, the value of *c(z, t)* can be obtained. Then the value of *c(z, t)* was substituted into Equation (7), thus the diffusion coefficient *D* was obtained.


$$loglog\left(\left|G^{*}\right|\right)=c_{1}loglog\left({\left|G^{*}\right|}_{1}\right)+c_{2}loglog\left({\left|G^{*}\right|}_{2}\right)$$ (8)

Where: $\left|G^{*}\right|$---G* values of the composite material, pa; ${\left|G^{*}\right|}_{1}$、${\left|G^{*}\right|}_{2}$--- G* values of the two materials respectively, pa; *c*_*1*_、*c*_*2*_---concentration or mass ratio of two materials.

#### 2.1.5. Establishment process of UV aging kinetics equation for asphalt.

Aging will cause the change of asphalt’s properties. Based on the theory of aging kinetics, establishing the aging kinetics equation for asphalt can help to predict its aging properties, and further analyze the change rate of asphalt’s properties, which helps to deeply reveal the decay mechanism of asphalt properties under different aging conditions.

The establishment process of aging kinetics equation for asphalt is mainly based on the theory of thermal analyzes kinetics. Expression of the aging kinetics equation is shown in Equation (9).


$$-\frac{dx}{dt}=k{\left[x(t)\right]}^{n}$$ (9)

Where: *x*(t)---properties of asphalt at t time; *k*---reaction rate constant; *n*---reaction order.

The key steps for establishing the aging kinetics equation for asphalt is to determine the reaction rate constant and reaction order. Experience has shown that for all simple reactions and most complex reactions, their reaction rate equation can be expressed in the form of Equation (10).


$$r=k{\left[C_{A}\right]}^{p}{\left[C_{B}\right]}^{q}$$ (10)

Where: *r*---reaction rate at t time; *C*_*A*_---concentration of reactant A at t time; *k*---reaction rate constant; p,q---reaction order; *C*_*B*_---concentration of reactant B at t time.

To determine the reaction rate constant and reaction order, referring to study of Ge [30] et al., the “planning and solving function” of EXCEL software will be utilized to find the suitable reaction order and reaction rate constants in this study. And the main calculation steps are described as follows:

(1) Integrate the Equation (10) to obtain its general solution equation:


$$\ln(x)=lnx_{0}-kt\;(n=1)$$ (11)

$$x^{1-n}=x_{0}^{1-n}+(n-1)kt\;(n\neq 1)$$ (12)

(2) Rewrite the Equation (11) and (12) to obtain their linear expressions:


$$x=x_{0}*exp(-kt)\;(n=1)$$ (13)

$$x={\left[x_{0}^{1-n}+(n-1)kt\right]}^{1/(1-n)}\;(n\neq 1)$$ (14)

The Equation (13) and (14) are the two nonlinear equations with parameters *n* and *k*, which can be solved by measuring the properties’ parameters of asphalt at different aging times. To make the calculation results more accurate, the selected time points are usually larger than two groups, that is, the selected experimental data groups are listed as follows: $\left\{\left(t_{0},x_{0}\right);\left(t_{1},x_{1}\right);\left(t_{2},x_{2}\right);\cdots \left(t_{i},x_{i}\right)\right\}$.

(3) Using the least square method to solve the above equations. Specifically, first, give initial values for parameters *n* and *k*; and then substitute the test data to Equation (13) or (14). Thus, the calculated values $x_{i}^{C}$ can be obtained. Equation (15) shows the calculation formula of squared relative deviations *P* between the test data values of asphalt’s properties *x*_*i*_ and the calculated values $x_{i}^{C}$.


$$\begin{array}{c} P=\sum {\mathrm{\backslash nolimits}}_{i=1}^{m}{\left((x_{i}-x_{i}^{C})/x_{0}\right)}^{2} \end{array}$$ (15)

Where: *x*_*i*_---the test data values of asphalt’s properties; $x_{i}^{C}$---the calculated values of asphalt’s properties.

The closer the *P* value is to 0, the more reasonable the calculation results of parameters *n* and *k* are. However, it is difficult to achieve *P* = 0. In this study, the mathematical iteration method was used to calculate the extreme value of *P*. And this step can be achieved through the “planning and solving function” of EXCEL software. Generally, it is required that *x*_0 _> *x*_*i*_ (*i* = 1,2,…), and *P* << 1.

### 2.3. Technical scheme

**Fig 6:**
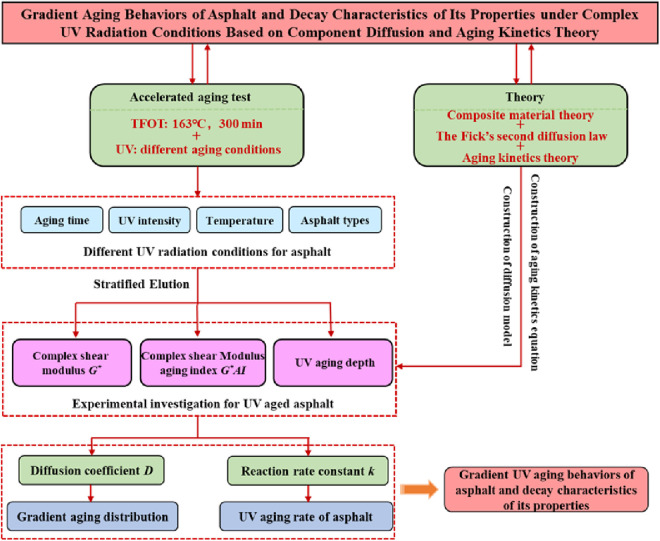
Experimental flow diagram.

Purpose of this study is to explore the decay characteristics of asphalt properties under different UV radiation conditions from the macro from the macro perspective. G* values of asphalt at different layers were measured, and then decay characteristics of asphalt’s properties under different UV aging conditions were analyzed by calculating the diffusion coefficient *D* and reaction rate constant *k*. The technical scheme of this study is shown in Fig 6.

## 3. Results and discussion

### 3.1. The variation characteristics of asphalt’s G* values

#### 3.1.1. G* values of aged asphalt under different UV aging times.

**Fig 7:**
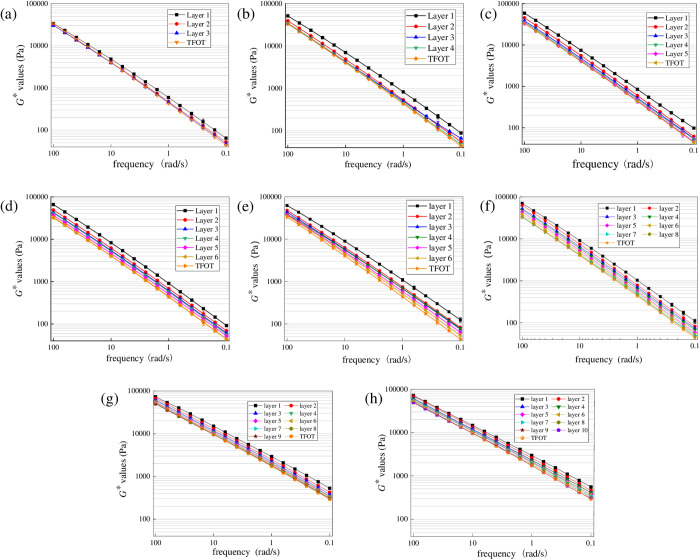
Variation characteristics of G* for SK70 at different aging times. **(a)** UV-3 h; **(b)** UV-6 h; **(c)** UV- 9 h; **(d)** UV- 12 h; **(e)** UV- 24 h; **(f)** UV- 72 h; **(g)** UV-144 h; **(h)** UV-216 **h.**

**Fig 8:**
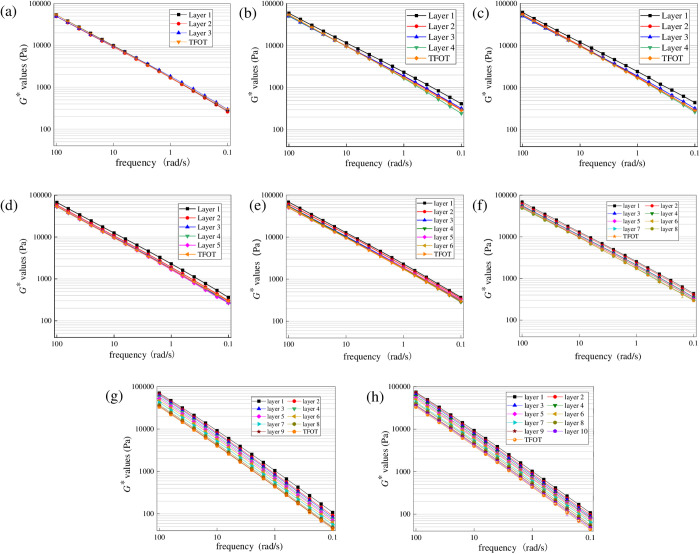
Variation characteristics of G* for SK70/SBS at different aging times. **(a)** UV-3 h; **(b)** UV-6 h; **(c)** UV- 9 h; **(d)** UV- 12 h; **(e)** UV- 24 h; **(f)** UV- 72 h; **(g)** UV-144 h; **(h)** UV-216 **h.**

To explore the diffusion depth of the surface UV aged asphalt with radiation times, aged asphalt under different radiation times was first peeled off layer by layer with trichloroethylene solutions. And then G* values were obtained. To control the uniqueness of variables, the aging temperature was set as 40°C and radiation intensity was set as 130 W/m^2^. And the results are shown in Fig 7 and Fig 8.

As shown in Fig 7, under different UV aging times, G* values of asphalt at different layers show an obvious gradient trend. And G* values of asphalt at the surface layer are significantly higher than those of other layers. Generally, when G* curve of asphalt at a certain layer is obviously separated from that of asphalt after TFOT aging, it can be considered that the UV aged asphalt at the surface of asphalt film has diffused to this layer. In other words, thickness of the film corresponding to this layer is the UV aging depth of asphalt. According to Fig 7, when the aging time is 3 h, only G* values of asphalt at the surface layer have increased compared to TFOT aged asphalt, while the G* curves at the second and third layers almost overlap, which indicates only the asphalt at surface layer will undergo the direct aging process under the ultraviolet radiation. When the aging time reaches to 6 h, G* values of asphalt at the second layer have a slight increase compared to TFOT aged asphalt, which indicates that asphalt at the surface layer has begun to diffuse at this time. When the aging time reaches to 9 h, G* curve of asphalt at the third layer has significantly separated from that of TFOT aged asphalt, indicating that UV aging depth of SK70 asphalt may have reached to the third layer. And when UV aging time continues to increase to 24 h, G* curve of asphalt at the sixth layer has significantly separated, which indicates that the UV aging depth has reached to the sixth layer.

Similar to the above phenomenon, when aging time reaches to 72 h, G* curve of asphalt at the seventh layer has significantly separated from that TFOT aged asphalt, showing that action depth of UV aging on asphalt may have reached about the seventh layer. And when the aging time reaches to 144 h, G* curve of asphalt at the eighth layer has significantly separated from that TFOT aged asphalt, while the G* curve of asphalt at the ninth layer almost overlaps with that TFOT aged asphalt, which indicates that UV aging depth of SK70# asphalt may have reached the ninth layer. Finally, when UV aging time continues to increase to 216 h, G* values of asphalt at the tenth layer slightly increase. Therefore, it can be determined that the UV aging depth of SK70 asphalt may has reached to the tenth layer.

Fig 8 shows the variation characteristics of G* for SK70/SBS at different aging times. According to Fig 8, when the aging time is 3 h, G* curves of asphalt at the stripped three layers almost overlap with those of TFOT aged asphalt, which indicates that the surface of SBS modified asphalt has not undergone the UV aging phenomenon. When the aging time reaches to 6 h, only G* curve of asphalt at the surface layer has separated from that of TFOT aged asphalt, while G* curves of asphalt at the second layer to fourth layer still overlaps with that of TFOT aged asphalt, which demonstrates that effect of UV aging on SBS modified asphalt only reaches the second layer. When the aging time increases to 9 h, the variation trend of G* curves of asphalt at different layers is similar to that under the UV aging time at 6 h, indicating that the aged asphalt at surface layer has not diffused obviously. And UV aging time increases to 24 h, G* values of asphalt at the second layer further increase, and gradually approach the G* values of asphalt at the surface layer; meanwhile, the G* curve of asphalt at the third layer gradually separates from the that of TFOT aged asphalt, which indicates that the diffusion depth of the surface UV aged asphalt further increase and may have reached to the third layer. When aging time increases to 72 h, G* values of asphalt at the fifth layer have slightly increased compared to those TFOT aged asphalt, demonstrating that the UV aging depth may have reached to the fifth layer. Subsequently, when aging time increases to 144 h, G* values of asphalt at different layers gradually increase, which demonstrates that the diffusion degree of aged asphalt further intensifies. And finally, when UV aging time reaches to 216 h, G* values of SK70/SBS modified asphalt at the eighth layer increase slightly, while G* values at the ninth and tenth layers are basically consistent with those of TFOT aged asphalt. Thus it can be determined that the aging depth of asphalt may have reached to the eighth layer.

From the above analysis, it can be concluded that similar to base asphalt, UV irradiation will first cause significant aging on the film surface of SBS modified asphalt, and then the aged asphalt on the surface will continuously diffuse into the deeper layers of asphalt, causing the aging depth of asphalt to gradually deepen. However, compared to base asphalt, UV aging depth of SBS modified asphalt are relatively smaller. This may be due to that the three-dimensional network structure formed in SBS modified asphalt can effectively hinder the diffusion process of the surface aged asphalt [31]. In addition, it should be noted that considering the limitation of this journal’s layout, this section only displays the analysis results of SK70 base asphalt and SK70/SBS modified asphalt. And as for SK90 base asphalt and SK90/SBS modified asphalt, effects of UV aging on their properties also show the significant gradient change trend.

**Fig 9:**
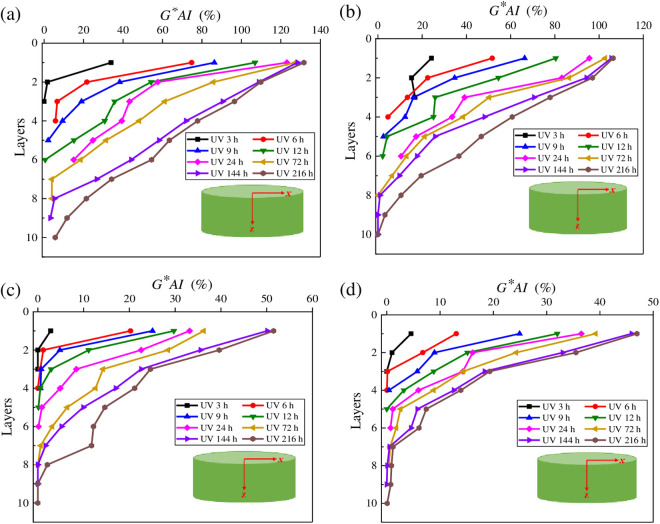
Variation characteristics of G*AI for different asphalt samples. **(a)** SK70; **(b)** SK90; **(c)** SK70/SBS; **(d)** SK90/SBS.


To intuitively illustrate the effect of radiation time on UV gradient aging behaviors of different types of asphalt, the complex shear modulus aging index G^*^AI of asphalt under the angular frequency of 10 rad/s was calculated. The calculation formula of G^*^AI is as shown in Equation (16). And the results are listed in Fig 9.

$$G^{*}AI=(G_{1}^{*}-G_{0}^{*})/G_{0}^{*}$$ (16)

Where: $G_{1}^{*}$--- the complex shear modulus of the asphalt samples before aging; $G_{0}^{*}$--- the complex shear modulus of the asphalt samples after aging.

As shown in Fig 9, under the same aging times, G*AI values of the four asphalt samples decrease in a nearly logarithmic form with the increasing of stripped layers; and when the stripped depth exceeds a certain value, the variation trend of G*AI values changes almost steadily until it finally approaches to zero, which indicates that the UV aging degree decreases continuously with the deepening of the stripping layer. In addition, based on the variation pattern of G*AI values, it can be inferred the UV aging depth of asphalt. Specifically, when the G*AI value of asphalt approaches to 0, it can be considered that asphalt in this layer has not been affected by the ultraviolet radiation, and the peeling depth at this condition is the UV aging depth of asphalt. Therefore, according to Fig 9, under the same ultraviolet radiation times, ranking for the UV aging depth for the four types of asphalt is listed as: SK90/SBS < SK70/SBS < SK90 < SK70, which indicates that the UV aging depth of SBS modified asphalt is relatively small.

#### 3.1.2 G* values of aged asphalt under different UV radiation intensities.

**Fig 10:**
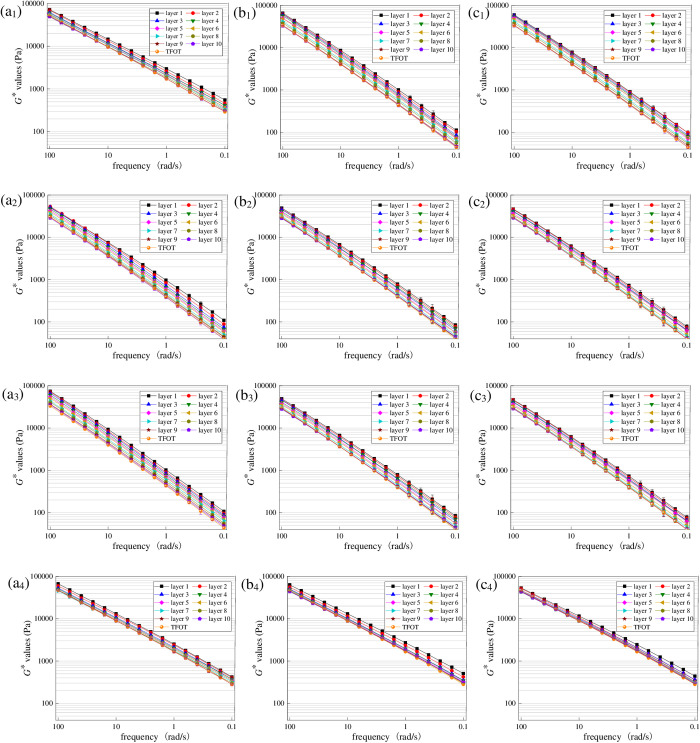
Variation characteristics of G* for asphalt under different radiation intensities. (a_1_) SK70−130 W/m^2^; (b_1_) SK70−110 W/m^2^; (c_1_) SK70−90 W/m^2^; (a_2_) SK90−130 W/m^2^; (b_2_) SK90−110 W/m^2^; (c_2_) SK90−90 W/m^2^; (a_3_) SK70/SBS-130 W/m^2^; (b_3_) SK70/SBS-110 W/m^2^; (c_3_) SK70/SBS-90 W/m^2^; (a_4_) SK90/SBS-130 W/m^2^; (b_4_) SK90/SBS-110 W/m^2^; (c_4_) SK90/SBS-90 W/m^2^.

To explore the effect of UV radiation intensity on UV gradient aging behavior of asphalt, aged asphalt under different radiation intensities was first peeled off layer by layer with trichloroethylene solutions, and then G* values were obtained. To control the uniqueness of variables, aging temperature was set as 40°C and UV aging time was set as 216 h in this section. Test results are shown in Fig 10.

As shown in Fig 10, under the radiation intensities of 130 W/m^2^, 110 W/m^2^ and 90 W/m^2^, G* curves of the four asphalt samples show a clear layering trend with the change of stripping layers, which further proves the significant gradient aging phenomenon occurs in the UV aging process of asphalt. Moreover, with the reduction of UV radiation intensity, difference in G* values of asphalt stripped from different layers gradually decrease, demonstrating that the UV aging depth of asphalt is continuously decreasing.

**Fig 11:**
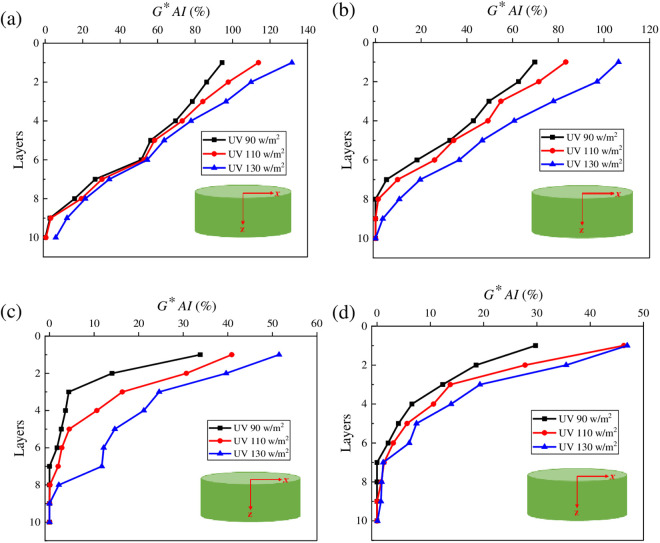
Variation characteristics of G*AI for asphalt under different radiation intensities. **(a)** SK70; **(b)** SK90; **(c)** SK70/SBS; **(d)** SK90/SBS.

To intuitively analyze the effect of UV radiation intensity on the UV gradient aging behaviors of asphalt, G*AI values of asphalt at different layers were calculated. And the results are shown in Fig 11.

As shown in Fig 11, with the increase of radiation intensities, G*AI values of the stripped asphalt under different layers gradually increase, indicating that the UV aging degree of asphalt continues to deepen. Meanwhile, compared with the variation rate in G*AI values of asphalt under the deeper layers, effect of radiation intensity on G*AI values of asphalt at the surface layer is greater. Through calculation, compared with the radiation intensity of 90 W/m^2^, G*AI values of SK70 asphalt at the surface layer increase by 20.53% and 39.43% under the radiation intensities of 110 W/m^2^ and 130 W/m^2^ respectively. Similarly, G*AI values of SK90 asphalt increase by 19.48% and 52.57%, G*AI values of SK70/SBS modified asphalt increase by 20.81% and 52.16%, and G*AI values of SK90/SBS modified asphalt increase by 55.69% and 57.87% respectively. The above results indicate that UV aging will significantly reduce the properties of asphalt film at the surface layer, and the higher the radiation intensity, the more serious the aging degree of asphalt.

Besides, based on the variation characteristics of G* and G*AI values of asphalt with the stripping depth, the UV aging depth of asphalt can be further inferred. When the radiation intensity is 130 W/m^2^, the UV aging depths of the four asphalt samples are listed as follows: SK70 asphalt (tenth layer), SK90 asphalt (ninth layer), SK70/SBS modified asphalt (eighth layer) and SK90/SBS modified asphalt (seventh layer). When the radiation intensity is 110 W/m^2^, the UV aging depths of the four asphalt samples are listed: SK70 asphalt (ninth layer), SK90 asphalt (eighth layer), SK70/SBS modified asphalt (eighth layer) and SK90/SBS modified asphalt (seventh layer). While when the radiation intensity is 90 W/m^2^, the UV aging depths of four asphalt samples are listed: SK70 asphalt (ninth layer), SK90 asphalt (eighth layer), SK70/SBS modified asphalt (seventh layer) and SK90/SBS modified asphalt (seventh layer). The above results indicate that during the UV aging process of asphalt, reduction of the radiation intensity can lead to UV aging depth of asphalt continuously decrease, but the impact degree is relatively small.

#### 3.1.3. G* values of aged asphalt under different aging temperatures.

**Fig 12:**
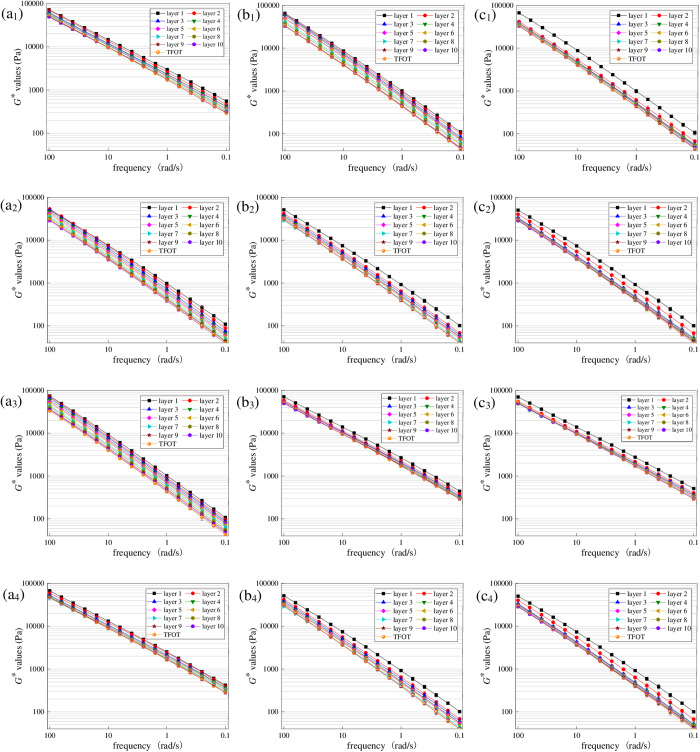
Variation characteristics of G* for asphalt under different aging temperatures. (a_1_) SK70−40°C; (b_1_) SK70−35°C; (c_1_) SK70−30°C; (a_2_) SK90−40°C; (b_2_) SK90−35°C; (c_2_) SK90−30°C; (a_3_) SK70/SBS-40°C; (b_3_) SK70/SBS-35°C; (c_3_) SK70/SBS-30°C; (a_4_) SK90/SBS-40°C; (b_4_) SK90/SBS-35°C; (c_4_) SK90/SBS-30°C.

To explore the effect of aging temperature on UV gradient aging behavior of asphalt, aged asphalt under different aging temperatures was first peeled off layer by layer, and then G* values were obtained. The radiation intensity was set as 130 W/m^2^ and UV aging time was set as 216 h in this section. Test results are shown in Fig 12.

**Fig 13:**
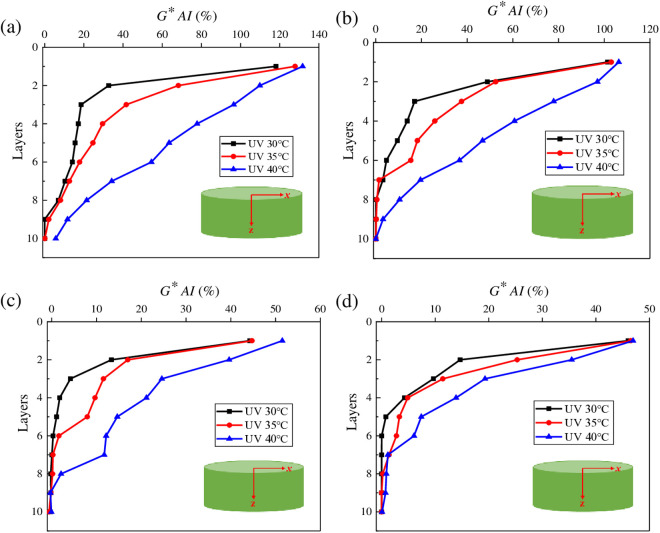
Variation characteristics of G*AI for asphalt under different aging temperatures. **(a)** SK70; **(b)** SK90; **(c)** SK70/SBS; **(d)** SK90/SBS.

As shown in Fig 12, under the different temperature conditions, G* values of the four asphalt also show a clear gradient distribution with the change of stripping layers. Besides, as the aging temperature changes, G* curves of asphalt stripped from the first layer doesn’t change a lot, which demonstrates that compared with UV radiation intensity, effect of aging temperature on UV aging degree of asphalt at the surface layer is relatively smaller. However, temperature has significant effect on the diffusion process of the surface aged asphalt. According to Fig 13, when aging temperature decreases from 40°C to 30°C, difference between the G* values of asphalt stripped from the second layer and those from the surface layer gradually increases, which manifests that as the aging temperature decreases, diffusion degree of the surface aged asphalt is reduced.

To intuitively analyze the effect of aging temperature on the UV gradient aging behavior of asphalt, G*AI values of asphalt at different layers were calculated. And the results are shown in Fig 13.

As shown in Fig 13, under the three aging temperature conditions, there is little difference in G*AI values of asphalt at the first layer, which indicates that aging temperature may not be the main factor that accelerating the UV aging of asphalt at the surface layer. However, temperature will affect the diffusion process of the aged asphalt. According to Fig 12 and Fig 13, the UV aging depth of asphalt can be inferred, and the values are listed as follows. When the aging temperature is 40°C, UV aging depth of the four asphalt samples are: SK70 asphalt (tenth layer), SK90 asphalt (ninth layer), SK70/SBS modified asphalt (eighth layer) and SK90/SBS modified asphalt (seventh layer). When the aging temperature is 35°C, the aging depths are: SK70 asphalt (ninth layer), SK90 asphalt (seventh layer), SK70/SBS modified asphalt (sixth layer) and SK90/SBS modified asphalt (seventh layer). And when the aging temperature decreases to 30°C, the aging depths change to: SK70 asphalt (ninth layer), SK90 asphalt (sixth layer), SK70/SBS modified asphalt (fourth layer) and SK90/SBS modified asphalt (fourth layer). The above results indicate that reduction of temperature will lead to UV aging depth of asphalt decrease obviously. The main reason is that temperature will affect the movement rate of the aged asphalt’s molecules. As the temperature decreases, the movement rate of the aged asphalt’s molecules on the surface layer slows down, which further leads to its diffusion degree towards deeper layers decrease.

### 3.2. Effect of radiation conditions on the UV aging depth for asphalt

To further explore the effect of radiation conditions on UV aging depth of asphalt, variation curves of aging depth under different UV aging conditions were plotted. And the specific steps are described as follows: first, according to the results of G* and G*AI values of asphalt peeled off from different layers, find the maximum diffusion layer of the aged asphalt. Then, weigh the quality of asphalt stripped off from each layer, and calculate the film thickness. Finally, the UV aging depth of asphalt can be obtained by adding up the above film thickness.

#### 3.2.1. UV aging depths for different types of asphalt.

**Fig 14:**
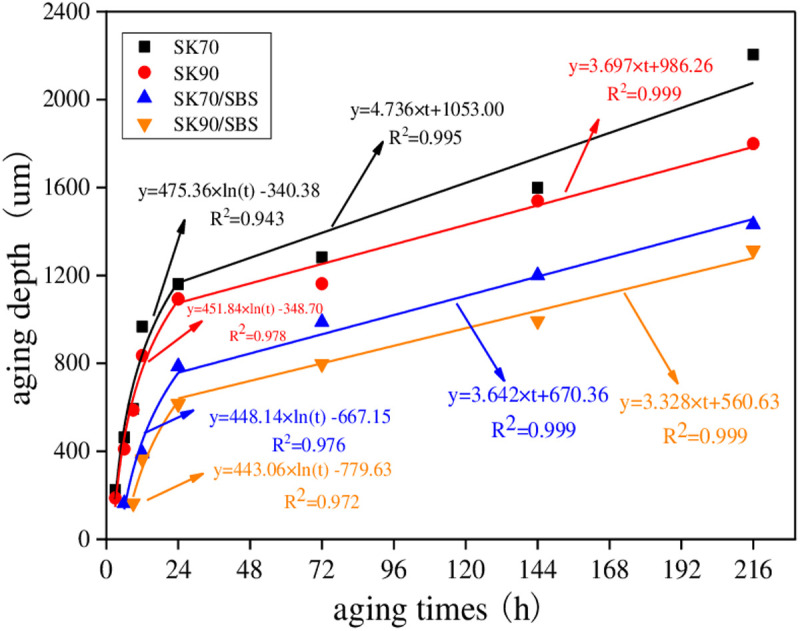
UV aging depths for different types of asphalt.

To analyze the effect of asphalt’s types on its UV aging diffusion process, UV aging depths for different types of asphalt were calculated. And to control the uniqueness of variables, aging temperature was set as 40°C and UV radiation intensity was set as 130 W/m^2^ in this section. Test results are shown in Fig 14.

As shown in Fig 14, under the same UV aging times, ranking for the aging depths of the four asphalt samples is listed as follows: SK70 > SK90 > SK70/SBS > SK90/SBS, which indicates that UV aging depth of SBS modified asphalt is smaller than that of base asphalt. And the above phenomenon also reflects that UV aging resistance of SBS modified asphalt is better than that of base asphalt indirectly. Meanwhile, UV aging resistance of asphalt with high grade is better than that of asphalt with low grade. This may be due to that content of aromatic in asphalt with high grade is relatively higher than that of asphalt with low grade.

Moreover, according to Fig 14, it can also be found that with the extension of aging time, UV aging depths of the four asphalt both gradually increase, but the increasing rate is closely related to aging times, which is similar to the research results of Li et al [25]. In the early stage of UV aging process (mainly referring to the aging time less than 24 hours), increasing rate of UV aging depth for asphalt is relatively large, which mainly increases in the form of logarithm model. However, when the irradiation time exceeds 24 h, increasing rate of UV aging depth for asphalt gradually slows down, which mainly increases in the form of linear model. Based on this, taking the UV aging time of 24 h as a cut-off point, a two-stage model (as shown in Equation 17) was established to characterize the changing process of UV aging depth for asphalt.


$$Z=\left\{ \begin{array}{c} a\times \ln(t)+b\;0\;h<t\leq 24\;h\\ c\times t+d\;t>24\;h\; \end{array}\right.\;$$ (17)

Where: *Z*--- UV aging depth of asphalt, um; *t*--- irradiation time, h; *a*, *b*, *c*, *d*---the regression coefficient.

**Fig 15:**
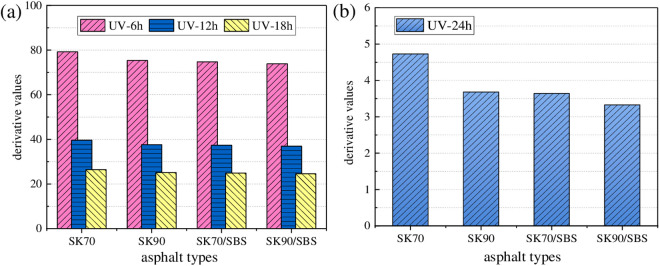
Change rate of the asphalt UV aging depth. (a) the first stage; (b) the second stage.

The above models are fitted through Origin software, and regression parameters are shown in Fig 14. It can be seen that there is a high correlation between UV aging depth of asphalt and UV aging times, and the two-stage models can characterize this relationship well. In addition, the change rate curves of the above models can be obtained by taking the first derivative of the model, which can be used to characterize the change rate of the UV aging depth for asphalt. To analyze the differences in UV aging depth between the four asphalt samples, derivative values of the above models under the UV aging times of 6 h, 12 h, 18 h and 24 h were calculated respectively. And results are shown in Fig 15.

As shown in Fig 15, under the same radiation times, ranking of derivative values for the four asphalt samples are listed as follows: SK70 > SK90 > SK70/SBS > SK90/SBS. The above results also illustrate that UV aging resistance of SBS modified asphalt is better than that of base asphalt, and that of asphalt with high grade is better than asphalt with low grade. Moreover, with the extension of radiation times, derivative values for the four asphalt both gradually decrease, and the derivative values in the second stage are significantly lower than those in the first stage, which illustrates that the diffusion process of surface aged asphalt mainly occurs in the early stage of UV aging process.

#### 3.2.2. UV aging depths for asphalt under different radiation intensities.

**Fig 16:**
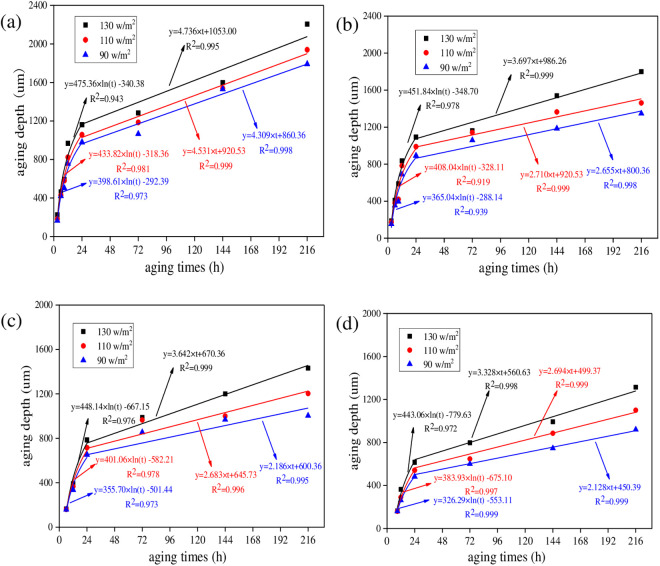
UV aging depth of asphalt under different irradiation intensity. **(a)** SK70; **(b)** SK90; **(c)** SK70/SBS;(d) SK90/SBS.

To analyze the effect of radiation intensity on UV aging depth of asphalt, UV aging depths of asphalt under different radiation intensities were calculated. The aging temperature was set as 40°C. And the results are shown in Fig 16.

**Fig 17:**
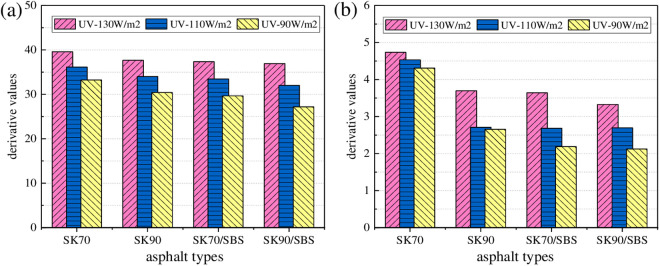
Change rate of the asphalt UV aging depth. **(a)** 12h; **(b)** 48h.

As shown in Fig 16, with the increase of radiation intensity, UV aging depth of the four asphalt both gradually increase, and the growth behavior can also be characterized by the logarithm model and linear model respectively. Similarly, derivative values of asphalt under different radiation intensities were calculated. And to make the data more representative, aging time was selected as 12 h and 48 h, which represents the first stage and second stage respectively. Test results are shown in Fig 17.

As shown in Fig 17, under same aging times, derivative values of the four asphalt gradually decrease with the reduction of radiation intensity, which indicates that radiation intensity will affect the asphalt UV aging depth to a certain extent. The reasons may lie in two aspects: on one hand, with the reduction of radiation intensity, aging degree of asphalt in the surface layer will decrease, thus its chemical composition will not change a lot compared with the TFOT aged asphalt. Further, the concentration difference of the chemical components between the surface and deep layers’ asphalt will also decrease, which will result in the decrease of the diffusion rate for the surface aged asphalt. On the other hand, the light energy possessed by ultraviolet light will also be converted into some thermal energy. And change of the radiation intensity will also indirectly affect the surface temperature of asphalt. Meanwhile, temperature is one of the main factors that affect the diffusion rate of asphalt. Thus, reduction of the radiation intensity will also cause the decrease in the surface temperature of asphalt, which further lead to the decrease in the diffusion rate of UV aged asphalt.

#### 3.2.3. UV aging depths for asphalt under different aging temperatures.

**Fig 18:**
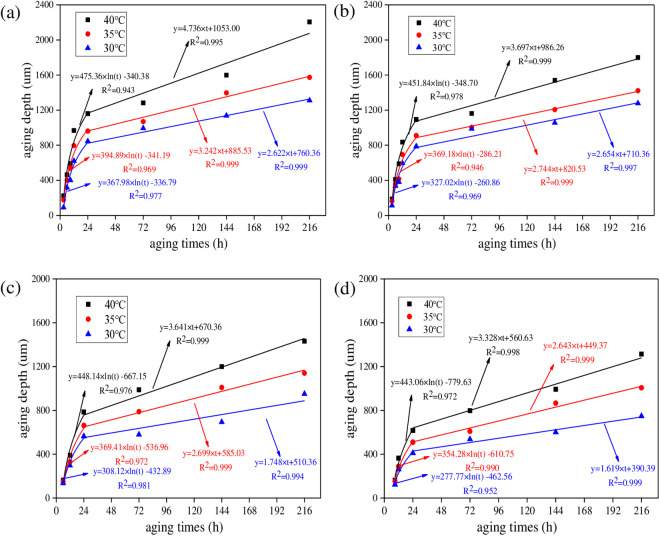
UV aging depth of asphalt under different aging temperature. **(a)** SK70; **(b)** SK90; **(c)** SK70/SBS;(d) SK90/SBS.

To analyze the effect of aging temperature on the UV aging depth of asphalt, UV aging depths of asphalt under different temperature were calculated. The radiation intensity was set as 130 W/m^2^. And the results are shown in Fig 18.

**Fig 19:**
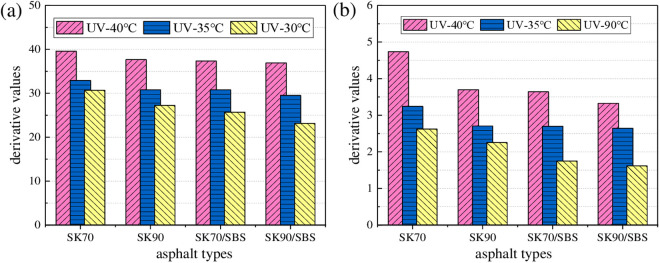
Change rate of the asphalt UV aging depth. **(a)** 12h; **(b)** 48h.

As shown in Fig 18, with the increase of aging temperature, UV aging depths of the four asphalt both gradually increase, which demonstrates that UV aging degree of asphalt becomes more serious. Similarly, the growth behavior of asphalt UV aging depth can also be fitted by the established two-stage models. To intuitively analyze the effect degree of aging temperature on UV aging depth of asphalt, derivative values were calculated, and the results are shown in Fig 19.

As shown in Fig 19, with the reduction of aging temperature, derivative values of the four asphalt both gradually decrease, which demonstrates that diffusion rate of the surface aged asphalt decrease. Moreover, compared with the radiation intensity, effect of aging temperature on the change rate of aging depth for asphalt is relatively higher. Taken the radiation times of 12 h as an example, when the radiation intensity decreases from 130 W/m^2^ to 90 W/m^2^, derivative values of the four asphalt decrease by 16.15% (SK70), 19.21% (SK90), 20.63% (SK70/SBS) and 29.36% (SK90/SBS), respectively. However, when aging temperature decreases from 40°C to 30°C, derivative values of the four asphalt decrease by 22.59% (SK70), 27.62% (SK90), 31.24% (SK70/SBS) and 37.30% (SK90/SBS), respectively. Reasons may be that: the higher the aging temperature, the more intense the movement of the aged asphalt molecules, and the faster the migration speed of the asphalt molecules. Therefore, diffusion rate of the surface UV aged asphalt will become faster.

From the above analysis, radiation intensity and aging temperature will both affect the UV aging depth of asphalt to a certain extent. To analyze the impact degree of the above factors on the UV aging depth of asphalt, variance analysis on derivative values were carried out. And results are shown in Table 3. It can be seen that the F ratio is greater than 1, which indicates that effect of aging temperature on the UV aging depth of asphalt is higher than that of radiation intensity. In addition, when UV aging time achieves to 48 h, the significance P value of radiation intensity is 0.157, which is obviously higher than 0.05, indicating that radiation intensity has less significant effect on the UV aging depth of asphalt at this time.

**Table 3:** Results of variance analysis.

Indicators	Sources	Squares	Freedom	Mean square	F	Significance	F ratio
**UV-12 h**	**Aging temperature**	172.308	2	86.154	8.245	.004	1.43
	**Radiation intensity**	120.647	2	60.324	5.773	.014	
	**Experimental error**	156.733	15	10.499	–	–	–
	**Total error**	358.880	19	–	–	–	–
**UV-48 h**	**Aging temperature**	5.793	2	2.896	5.479	.016	2.62
	**Radiation intensity**	2.217	2	1.108	2.093	.157	
	**Experimental error**	7.929	15	0.529	–	–	–
	**Total error**	13.955	19	–	–	–	–

### 3.3. Calculation results of diffusion coefficient *D*

To explore the differences in diffusion rate of the surface aged asphalt under different UV aging test conditions, diffusion coefficient *D* of surface aged asphalt at different stripped layers was calculated according to the mentioned steps in the section of 2.1.4. To simplify the solution process, it must be noted that the following assumptions need to be made during the calculation: the aged asphalt samples for the diffusion layer is regarded as a mixture cause by the co-diffusion of aged asphalt in the surface layer and that below the next layer.

#### 3.3.1. Diffusion coefficient of asphalt under different aging times.

**Fig 20:**
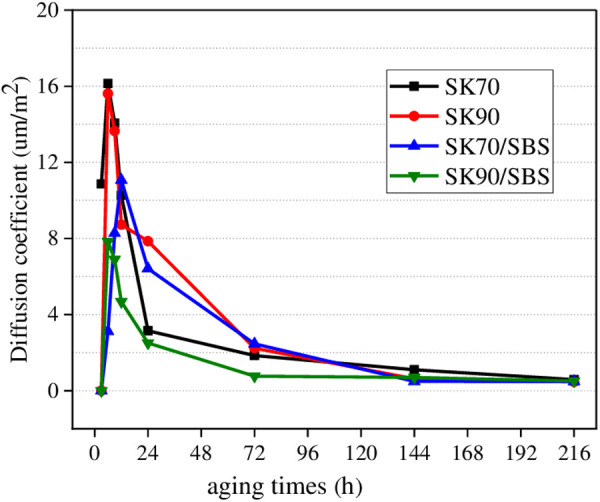
Diffusion coefficient of asphalt under different aging times.

*D* values of the surface aged asphalt in the second layer under different aging times were calculated. In this section, aging temperature was set as 40°C, and radiation intensity was set as 130 W/m^2^. Results are shown in Fig 20.

According to Fig 20, with the extension of aging time, *D* values of the four asphalt both show the trend of increasing first, then decreasing, and finally stabilizing. When the radiation time is shorter, *D* values of asphalt increase rapidly, indicating that diffusion rate of the surface aged asphalt is faster at this time. The reason is that once the asphalt film is irradiated by ultraviolet light, its chemical components will change rapidly, which further causes the concentration difference between the surface layer’s aged asphalt and the deep layer’s asphalt become larger, thus the diffusion rate of aged asphalt is fastest. However, as the aging time increases, a hard carbonized layer will be formed on the asphalt surface, which can effectively reflect some ultraviolet light [32]. Thus, aging rate of asphalt at the surface layer decreases, and the change degree of its chemical components also decreases. Besides, during the diffusion process of the surface aged asphalt to the deeper layer, asphalt in the deeper layer will also diffuse upward. In other words, there is a diffusion and fusion process between the aged asphalt at the surface layer and the new asphalt at the deeper layer. Under the above influence factors, concentration difference between the aged asphalt at surface layer and asphalt at deep layer will decrease, thus diffusion rate of the aged asphalt gradually decreases and finally approaches to a stable state.

#### 3.3.2. Diffusion coefficient of asphalt under different aging temperatures.

**Fig 21:**
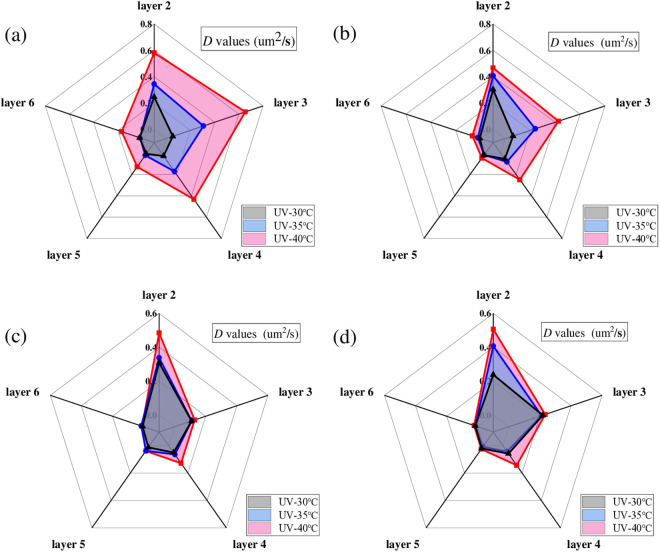
Diffusion coefficient of asphalt under different aging temperatures. **(a)** SK70; **(b)** SK90; **(c)** SK70/SBS;(d) SK90/SBS.

To analyze the effect of aging temperature on the diffusion process of the UV aged asphalt, *D* values of the surface aged asphalt at the first six layers were calculated respectively. In this section, radiation intensity was set as 130 W/m^2^, and aging time was set as 216 h. Results of *D* values are shown in Fig 21.

As shown in Fig 21, with the increasing of UV aging temperature, *D* values of the four asphalt at different layers both increase, which indicates that the diffusion rate of the surface aged asphalt gradually increases. Influence of temperature on the diffusion process mainly lies in its effect on the molecular motion rate of asphalt. The movement rate of the aged asphalt’s molecules will increase at high aging temperature; thus, diffusion rate of asphalt will accelerate, and *D* values of asphalt will become larger. Moreover, it can also be found that as the stripping layers deepen, *D* values of the surface aged asphalt gradually decrease, indicating that its diffusion degree in deeper layers decreases.

#### 3.3.3. Diffusion coefficient of asphalt under different radiation intensities.

**Fig 22:**
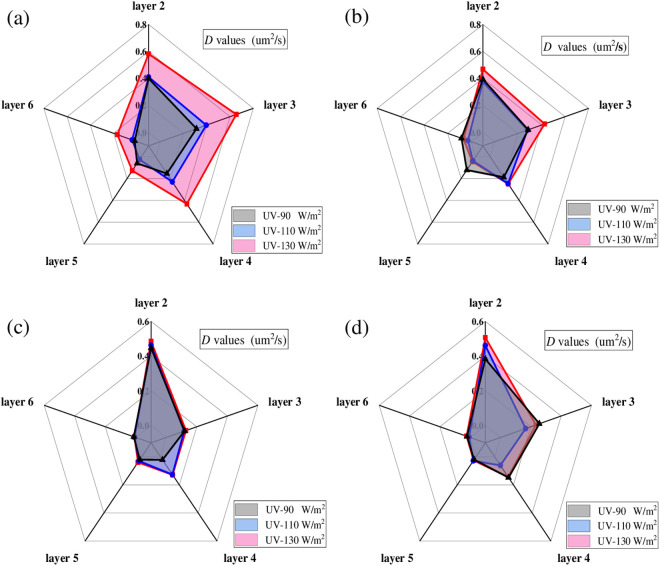
Diffusion coefficient of asphalt with different radiation intensity. **(a)** SK70; **(b)** SK90; **(c)** SK70/SBS;(d) SK90/SBS.

To analyze the effect of radiation intensity on the diffusion process of the UV aged asphalt, *D* values of the surface aged asphalt at the first six layers were also calculated respectively. In this section, the aging temperature was set as 40°C, and aging time was set as 216 h. Results of *D* values are shown in Fig 22.

As shown in Fig 22, with the increasing of radiation intensity, *D* values of the four asphalt samples at different layers also gradually increase, which indicates that increasing the ultraviolet radiation intensity can also accelerate the diffusion rate of surface aged asphalt. However, compared with the aging temperature, effect degree of radiation intensity on the *D* values of asphalt is relatively lower. Especially for SBS modified asphalt, it can be clearly seen that its diffusion coefficients almost do not change with the variety of radiation intensity.

### 3.4. Results of UV aging kinetics equation for asphalt

To further explore the effect of different UV aging test conditions on the decay of asphalt properties, taken the G* values of the asphalt at the surface layer as indicator, UV aging kinetics equations for asphalt under different aging test conditions were established according to the mentioned steps in the section of 2.1.5.

#### 3.4.1. UV aging kinetics equation for different types of asphalt.

**Fig 23:**
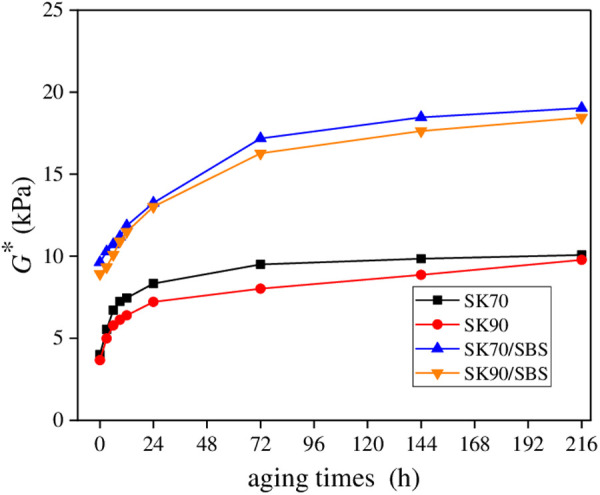
G* values of asphalt under different aging times.

To analyze the difference in UV aging process between different types of asphalt samples, G* values of the four asphalt samples at the surface layer were obtained. In this section, aging temperature was set as 40°C, and radiation intensity was set as 130 W/m^2^. The results are shown in Fig 23.

As shown in Fig 23, with the extension of UV aging times, G* values of the four asphalt both gradually increase, but the increasing rates gradually decrease in the later aging stage. According to the variation characteristics of G* values, the UV aging process of asphalt can be divided into two stages. The first stage is in the early period of ultraviolet radiation (mainly referring to the aging time less than 24 hours), during which G* values of asphalt show a rapid growth trend with the extension of aging times, and the UV aging rate of asphalt is relatively faster. The second stage is in the later period of ultraviolet radiation, during which G* values of asphalt tend to be stable with the extension of aging times, and the UV aging rate of asphalt gradually slows down. Therefore, it can be considered that the UV aging process of asphalt mainly occurs in the early radiation stage, which is consistent with the research conclusions in the previous sections. Further, UV aging kinetics equation of asphalt were established, and to make the calculation process more convenient, the 1/G* was set as the index. And results of each parameter are listed in Table 4.For the parameters in Table 4, the reaction rate constant *k* is commonly used to characterize the speed of a reaction, and the larger the *k* value, the faster the reaction rate. From Table 4, ranking of *k* values for the four asphalt is listed as follows: SK90/SBS < SK70/SBS < SK90 < SK70, which indicates that the UV aging rate of SBS modified asphalt is lower than that of base asphalt, thus SBS modified asphalt exits better UV aging resistance. Moreover, as for the base asphalt with different grades, asphalt with high grade exits better UV aging resistance than asphalt with low grade.

**Table 4:** Fitting results of UV aging kinetics equation for asphalt.

Asphalt types	*1/G* ^*^		
***n***	***k***	***P***
**SK70**	7	1860.58	0.007
**SK90**	7	1086.49	0.009
**SK70/SBS**	5	535.36	0.090
**SK90/SBS**	5	438.39	0.075

#### 3.4.2. UV aging kinetics equation of asphalt under different radiation intensities.

**Fig 24:**
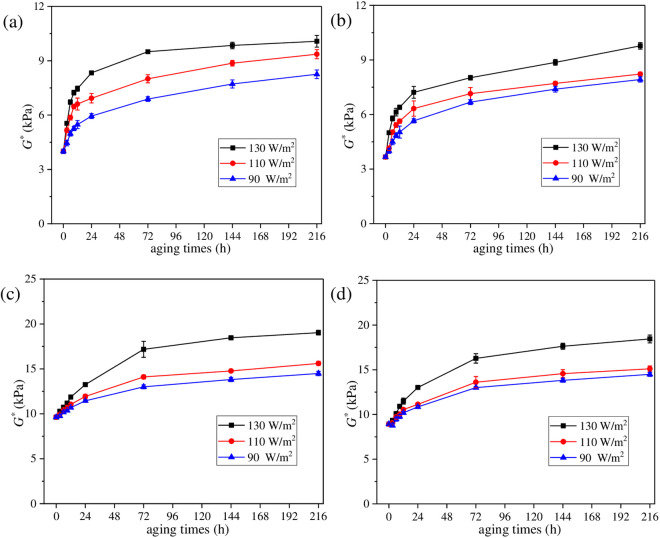
G* values of asphalt under different irradiation intensity. **(a)** SK70; **(b)** SK90; **(c)** SK70/SBS;(d) SK90/SBS.

To analyze the effect of radiation intensity on UV aging process of asphalt, taken the aging temperature of 40°C as an example, G* values of asphalt under different radiation intensity were obtained. And the results are shown in Fig 24.

As shown in Fig 24, under the same aging times, G* values of the four asphalt both gradually increase with the increase of radiation intensity, demonstrating that aging degree of asphalt becomes more serious. Similarly, UV aging kinetics equations of asphalt under different radiation intensity were established, and the results of each parameter are listed in Table 5. From Table 5, it can be seen that with the increasing of UV radiation intensity, *k* values of the four asphalt both gradually increase, indicating that the UV aging rate of asphalt accelerates. The reason may be that enhancement of UV radiation intensity will promote the increasing of light energy on the surface of asphalt film. Thus, the rate of chemical bonds inside asphalt molecules to enter the excited state will be accelerated. And in the presence of oxygen, the above chemical bonds will undergo the oxidation reduction reactions rapidly, which will lead to an increasing of polar oxygen-containing functional groups within asphalt [31]. Therefore, the decay rate of asphalt’s properties increases.

**Table 5:** Fitting results of UV aging kinetics equation for asphalt.

Types	Irradiation intensity	Parameters			Types	Irradiation intensity	Parameters		
		*n*	*k*	*P*			*n*	*k*	*P*
**SK70**	130 W/m^2^	7	1860.58	0.0073	**SK70** **/SBS**	130 W/m^2^	5	535.36	0.0900
	110 W/m^2^	7	855.81	0.0056		110 W/m^2^	5	89.90	0.0084
	90 W/m^2^	7	209.26	0.0057		90 W/m^2^	5	58.20	0.0065
**SK90**	130 W/m^2^	7	1086.49	0.0090	**SK90** **/SBS**	130 W/m^2^	5	438.39	0.0750
	110 W/m^2^	7	320.12	0.0081		110 W/m^2^	5	81.52	0.0071
	90 W/m^2^	7	132.26	0.0060		90 W/m^2^	5	59.16	0.0076

#### 3.4.3. UV aging kinetics equation of asphalt under different aging temperatures.

**Fig 25:**
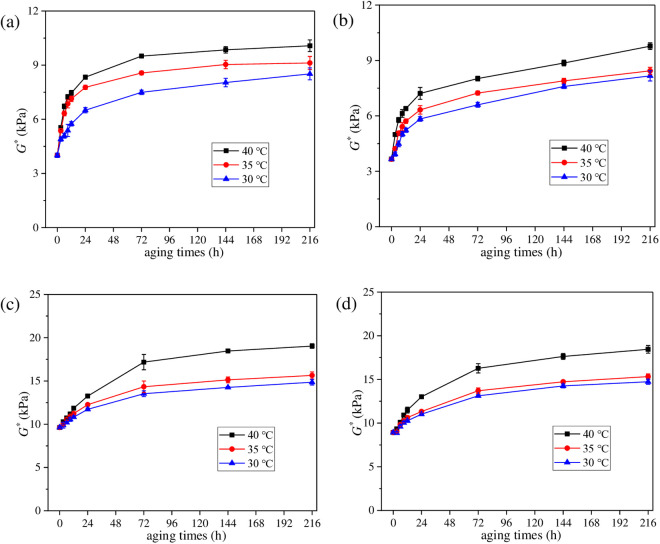
G* values of asphalt under different aging temperature. **(a)** SK70; **(b)** SK90; **(c)** SK70/SBS;(d) SK90/SBS.

Temperature is an important factor that affects the reaction rate of materials. To analyze the effect of aging temperature on UV aging process of asphalt, taken the radiation intensity of 130 W/m^2^ as an example, G* values of the four asphalt samples under different aging temperature were obtained. And the results are shown in Fig 25.

According to Fig 25., during the UV aging process, effect of aging temperature on G* values of asphalt is similar to that of radiation intensity. Specifically, under the same aging times, G* values of the four asphalt both gradually increase with the increase of aging temperature, demonstrating that aging degree of asphalt becomes more serious. Furthermore, in the first stage of UV aging process, the higher the aging temperature, the faster the growth rate of asphalt’s G* values, which indicates that the aging rate of asphalt accelerates. However, with the extension of aging time, effect degree of temperature on asphalt’s G* values decrease, which demonstrates that effect of temperature on UV aging rate of asphalt mainly occurs in the early stage of UV radiation. Similarly, UV aging kinetics equations of asphalt under different aging temperature were established, and the results of each parameter are listed in Table 6. From Table 6, it can be seen that with the increasing of aging temperature, *k* values of the four asphalt samples both gradually increase, which indicates that the UV aging rate of asphalt accelerates. The above phenomenon shows a consistent variation characteristic with the effect of UV radiation intensity on UV aging rate of asphalt.

**Table 6:** Fitting results of UV aging kinetics equation for asphalt.

Types	Irradiation intensity	Parameters			Types	Irradiation intensity	Parameters		
		*n*	*k*	*P*			*n*	*k*	*P*
**SK70**	40°C	7	1860.58	0.0073	**SK70** **/SBS**	40°C	5	135.36	0.0152
	35°C	7	1057.70	0.0132		35°C	5	102.23	0.0118
	30°C	7	406.45	0.0013		30°C	5	61.98	0.0094
**SK90**	40°C	7	486.49	0.0054	**SK90** **/SBS**	40°C	5	98.39	0.0109
	35°C	7	344.94	0.0058		35°C	5	88.82	0.0085
	30°C	7	144.41	0.0119		30°C	5	59.15	0.0093

#### 3.4.4. Main factor affecting the UV aging rate of asphalt.

**Fig 26:**
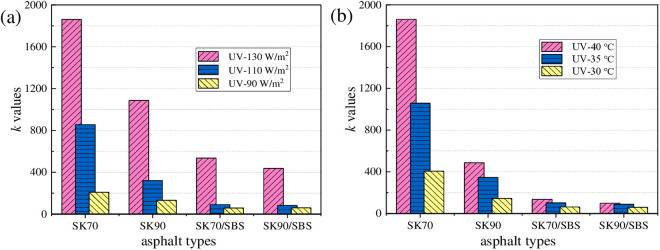
*k* values of the four asphalt samples. (a) Irradiation intensity; (b) Aging temperature.

From the above analysis, it can be concluded that UV radiation intensity and aging temperature will both affect the UV aging rate of asphalt to a certain extent. To intuitively analyze the effect degree of the above two factors on the UV aging rate of asphalt, *k* values of the four asphalt samples under different radiation intensity and aging temperature were compared, and the results are shown in Fig 26.

As shown in Fig 26, with the reduction of radiation intensity and aging temperature, the reaction rate constants *k* of the four asphalt gradually decreases, indicating that the UV aging degree of asphalt slows down, thus the decay rate of asphalt’s properties also decreases. To analyze the effect degree of the above factors on the reaction rate constant, SPSS software was used to perform variance analysis on the *k* values, and the results are shown in Table 7. It can be seen that aging temperature and radiation intensity both have a significant effect on the UV aging rate of asphalt. And by comparison, effect of radiation intensity is greater, which indicates the radiation intensity dominates the main effect in the degradation process of asphalt’s UV aging properties.

**Table 7:** Results of variance analysis.

Sources	Squares	Freedom	Mean square	F	Significance	F ratio
**Aging temperature**	1408141.98	2	704070.99	4.430	0.031	0.865
**Irradiation intensity**	1628839.40	2	814419.68/	5.124	0.020	
**Experimental error**	2384051.48	15	158936.77	–	–	–
**Total error**	4299744.33	19	–	–	–	–

#### 3.4.5. Analyze of decay mechanism of asphalt properties with UV aging.

From the above analysis, it can be found that an obvious two-stage reaction phenomenon exits in UV aging process of asphalt. The first stage is in the initial stage of UV irradiation, during which asphalt properties change rapidly with the non-linear trend, indicating that the UV aging rate of asphalt is relatively higher. The second stage is in the later stage of UV irradiation, during which asphalt properties change slowly with the extension of aging time. According to previous studies, under the strong UV irradiation, chemical bond in asphalt molecule will transform from ground state to excited state because of its absorption of energetic UV light, which further leads to the breaking of chemical bonds. And with the presence of oxygen, the broken chemical bonds will undergo the oxidative addition reactions, thus polar oxygen-containing functional groups, which further causes the decline of asphalt road properties. At the beginning of UV irradiation, due to the relatively large amount of chemical bonds that are easily excited in asphalt molecules, the rate of forming polar functional groups will be faster, which implies that UV aging rate of asphalt is faster. However, with the extension of UV aging times, the unstable chemical bonds that are easily excited in asphalt molecules gradually decrease, thus the UV aging rate of asphalt will become slow and eventually tend to be stable. Besides, it should be noted that during the initial irradiation period, the serious UV irradiation will also cause a carbonized hard layer appearing on asphalt film surface, which will hinder the properties deterioration caused by aging, thus reducing the UV aging rate of asphalt in the later stage [26,31].

In addition, aging temperature and radiation intensity both have a significant effect on the UV aging rate of asphalt. There is no doubt that temperature will accelerate the aging rate of asphalt, and the higher the aging temperature, the faster the UV aging rate of asphalt. Influence of radiation intensity on the reaction rate of asphalt UV aging mainly lies in the light energy carried by UV light. And higher radiation intensity means that a higher light energy projected onto the surface of asphalt sample per unit time, thus, the rate of chemical bonds in asphalt molecules reaching the excited state will become faster. Meanwhile, the light energy possessed by ultraviolet light will also be converted into some thermal energy, which further indirectly increase the surface temperature of asphalt, thus accelerating the UV aging rate. Compared with aging temperature, radiation intensity dominates the main effect in the degradation process of asphalt’s UV aging properties, which is also an important reason for the serious aging phenomenon of asphalt pavement in areas with severe UV radiation but relatively low temperature (such as Northwest China). And based on the analysis results of section 3.2.3, temperature will also have a significant impact on UV aging diffusion process of asphalt, which further leads to the increase of UV aging depth of asphalt. Therefore, at the high temperature seasons, aging phenomenon of asphalt pavement is more serious.

## 4. Conclusions

To explore the UV aging process of asphalt under different test conditions, asphalt film specimens were subjected to UV aging test under complex radiations for 1–216 h. After aging, the aged asphalt was stripped layer by layer to test rheological properties form different layers. On this basis, the UV gradient aging behaviors and UV aging depth of asphalt were analyzed with G*, G*AI and diffusion coefficient *D*, and the UV aging kinetics equations of asphalt under different aging conditions were established as well. The main conclusions can be drawn.

(1) After UV aging, the complex shear modulus G* of asphalt within a certain depth increases, and the established G* curves of asphalt show an obvious gradient distribution law, which indicates that UV aging process of asphalt exists the significant gradient aging behaviors.
(2) UV aging depth of asphalt can be obtained from the changing trends of G* values of asphalt, and it can be found that there is a high correlation between the UV aging depth of asphalt and the aging time, and the growth behavior can be characterized by a two-stage model. During the initial 0 ~ 24 h of UV aging, the increasing rate of asphalt’s UV aging depth is relatively higher, and its growth behavior accords with the logarithm model. When the aging times exceeds 24 h, the increasing rate of asphalt’s UV aging depth tends to be stable, which accords with the linear model.
(3) Under the same UV aging times, enhancement of irradiation and aging temperature can both lead to the increase of UV aging depth of asphalt, and by contrast, the effect degree of temperature on UV aging depth is larger. Besides, UV aging depth of SBS modified asphalt is smaller than base asphalt, and that of asphalt with high grade is also smaller than asphalt with low grade.
(4) To analyze the UV aging process of asphalt, UV aging kinetics of asphalt at the surface layer were established with the aid of G* index. It can be concluded that UV aging process of asphalt also follows the two-stage reaction phenomenon. The first stage is the rapid response stage, during which the asphalt properties show a non-linear and rapid decay trend with the extension of irradiation time. The second stage is the slow reaction stage, during which asphalt properties show a linear slow decay trend with the extension of irradiation time. Similarly, aging time of 24 hours can be set as the criteria to distinguish between the two-stage reaction zones.
(5) Under the same aging times, enhancement of radiation intensity and aging temperature can both lead to the increase in the reaction rate constant *k* of asphalt, indicating the UV aging rate of asphalt increases. And by contrast, radiation intensity has a greater influence on the aging reaction rate constant *k* of asphalt than temperature, which is different from their influence on UV aging depth.

Despite these promising results obtained in this study, challenges remain; the results should be confirmed through further research with some advanced micro testing instruments. Future researches are required to study the microstructure and chemical composition of asphalt with UV aging. Additionally, a correlation should be also established between the macro properties of UV aged asphalt and its chemical composition, so as to further analyze the UV aging mechanism of asphalt.
